# Clinical application of machine learning and computer vision to indocyanine green quantification for dynamic intraoperative tissue characterisation: how to do it

**DOI:** 10.1007/s00464-023-09963-2

**Published:** 2023-03-09

**Authors:** Niall P. Hardy, Pol MacAonghusa, Jeffrey Dalli, Gareth Gallagher, Jonathan P. Epperlein, Conor Shields, Jurgen Mulsow, Ailín C. Rogers, Ann E. Brannigan, John B. Conneely, Peter M. Neary, Ronan A. Cahill

**Affiliations:** 1 UCD Centre for Precision Surgery, School of Medicine, University College Dublin, Dublin, Ireland; 2 IBM Research Europe, Dublin, Ireland; 3 Department of General and Colorectal Surgery, Mater Misericordiae University Hospital, Dublin, Ireland; 4 Department of Hepatobiliary, Foregut and Bariatric Surgery, Mater Misericordiae University Hospital, Dublin, Ireland; 5 Department of General and Colorectal Surgery, University Hospital Waterford, University College Cork, Waterford, Ireland

**Keywords:** Fluorescence-guided surgery, Indocyanine green quantification, Colorectal cancer, Artificial intelligence, Machine learning

## Abstract

**Introduction:**

Indocyanine green (ICG) quantification and assessment by machine learning (ML) could discriminate tissue types through perfusion characterisation, including delineation of malignancy. Here, we detail the important challenges overcome before effective clinical validation of such capability in a prospective patient series of quantitative fluorescence angiograms regarding primary and secondary colorectal neoplasia.

**Methods:**

ICG perfusion videos from 50 patients (37 with benign (13) and malignant (24) rectal tumours and 13 with colorectal liver metastases) of between 2- and 15-min duration following intravenously administered ICG were formally studied (clinicaltrials.gov: NCT04220242). Video quality with respect to interpretative ML reliability was studied observing practical, technical and technological aspects of fluorescence signal acquisition. Investigated parameters included ICG dosing and administration, distance–intensity fluorescent signal variation, tissue and camera movement (including real-time camera tracking) as well as sampling issues with user-selected digital tissue biopsy. Attenuating strategies for the identified problems were developed, applied and evaluated. ML methods to classify extracted data, including datasets with interrupted time-series lengths with inference simulated data were also evaluated.

**Results:**

Definable, remediable challenges arose across both rectal and liver cohorts. Varying ICG dose by tissue type was identified as an important feature of real-time fluorescence quantification. Multi-region sampling within a lesion mitigated representation issues whilst distance–intensity relationships, as well as movement-instability issues, were demonstrated and ameliorated with post-processing techniques including normalisation and smoothing of extracted time–fluorescence curves. ML methods (automated feature extraction and classification) enabled ML algorithms glean excellent pathological categorisation results (AUC-ROC > 0.9, 37 rectal lesions) with imputation proving a robust method of compensation for interrupted time-series data with duration discrepancies.

**Conclusion:**

Purposeful clinical and data-processing protocols enable powerful pathological characterisation with existing clinical systems. Video analysis as shown can inform iterative and definitive clinical validation studies on how to close the translation gap between research applications and real-world, real-time clinical utility.

**Supplementary Information:**

The online version contains supplementary material available at 10.1007/s00464-023-09963-2.

Indocyanine green (ICG) fluorescence angiography can be used to assist in the delineation of surgical anatomy and assessment of tissue physiology as well as to identify malignant pathology with varying levels of accuracy [1–4]. Moving beyond surgeon visual interpretation, computer vision and machine learning (ML) methods can extract real-time dynamic time–fluorescence profiles of ICG inflow and outflow in tissues enabling mathematical examination and extrapolation. Such profiles can, thereby, be compared and categorised into clinically relevant determinants providing artificial intelligence (AI)-based insights into tissue nature. These and other approaches for ICG signal interpretation and quantification have been described in research applications for the assessment of colonic perfusion [5–9] and even the characterisation of rectal cancer vs benign neoplastic tumours [10–13].

However, concerns have been raised regarding the translation of such computerised quantitative ability into current NIR systems as such systems have been primarily designed as clinical tools for image creation and display for human interpretation and not precise computational measurement methods [11, 14, 15]. Previous work has demonstrated discrepancies in detected fluorescence intensities with ICG circulation (related to both dosing amount and methods as well as patient and anaesthesia-related variables) and further vary by on-screen tissue location (e.g. near vs far, centre vs periphery) [16–18]. Other potential pitfalls for interpretation by both humans and machines include the clinically observed phenomenon of ICG tissue diffusion vs true perfusion and the fact that rectal tumours can contain areas of admixed malignant and benign disease. [17, 19] Camera and tissue movement as well as any other impingement upon the field of view (e.g. by instrumentations) can also undermine accurate extrapolation of insights from dynamic imagery [17].

Given the growing interest and potential clinical utility of ML in the field of ICG fluorescence signal quantification, we sought to identify the relative importance of such considerations in a clinical series of fluorescence angiograms from patients with neoplasia and determine potential mitigating strategies on how useful these might be. Pertinent findings can then inform further endeavours to clinically characterise tissues by their ICG perfusion profiles with and without ML.

## Methods

### Patients and methods

Multispectral (white light and NIR) videos from 50 patients (including 37 with rectal tumours, 13 of which were benign and 24 malignant, and 13 colorectal liver metastases (CRLM)) capturing ICG inflow and early outflow over between 2 and 15 min of direct observation immediately following intravenously administered ICG were studied. All patients were undergoing diagnostic and therapeutic surgical interventions and were specifically consented for inclusion in prospective study (the ‘Future of Colorectal Cancer Surgery’ project, ClinicalTrials.gov NCT04220242, ethical approval ref: 1/378/2092). Videos were created and recorded using a commercial near-infrared (NIR) imager (Pinpoint Endoscopic Imaging System, Novadaq/Stryker Corp, Kalamazoo, MI, USA) with each patient receiving systemic ICG whilst the neoplastic area of interest was being observed continually as part of a defined protocol. Patients with rectal pathology (whether benign or malignant) had all been referred for surgical consideration by a medical gastroenterologist after initial colonoscopy and were examined endoscopically in lithotomy position under general anaesthesia as described previously [10]. All those with liver metastases were undergoing therapeutic resection either via a primary laparoscopic or open approach with the NIR camera again being deployed intraoperatively.

### Video analysis

Video recordings from these patients, as well as time–fluorescence curves previously generated using open-source fluorescence tracking software through tracking of expert annotated regions of interest (demarcating both healthy and unhealthy areas within the same screen view) within these videos [11, 20], were scrutinised to identify examples of potential discrepancies and pitfalls in their interpretation based on previous observations from the published literature in the field of fluorescence-guided surgery. These included examination for any impact of variation in ICG dosing on the detected fluorescence intensity values from all generated time–fluorescence curves. All studied rectal lesion interrogations had been performed using 0.25 mg/kg fixed dosing. The liver lesions included for study had been imaged with both 0.1 and 0.05 mg/kg of ICG with the lower dose being trialled due to signal saturation (pixel greyscale unit > 255) at the higher dose. Signal attenuation resulting from varying distance between camera and target as well as the impact of movement (both in the tissue and by the camera) were also assessed. Sampling bias, both in the post hoc user-selected region of interest (ROI) and in the observation period (video length), was also studied.

Once identified, methods to ameliorate such issues were developed and applied via clinical protocol standardisation (e.g. ICG dosing alteration and administration standardisation) and accepted ML mathematical methods (e.g. normalisation of time–intensity curves obtained by dividing every ROI intensity value by the curve’s peak intensity reading) and their mitigation impact studied. Computer generated curves, as well as clinically obtained curves, were created to demonstrate both the underlying principle and the relative impact of any methodological attempt at correction as follows where not otherwise explained above.

### Video acquisition challenges

Distance–intensity relationships and the degree of camera movement occurring were demonstrated clinically in real time in a patient with rectal cancer (post-neoadjuvant treatment) undergoing tumour assessment under general anaesthesia. This patient was placed in the lithotomy position with a tabletop electromagnetic (EM) tracking system (Aurora NDI, Canada) positioned under padding beneath their sacrum to include the anatomical ROI within the EM field. A motion sensor (measuring movement in the x, y and z axis along with rotation around the x and y axis) was secured to the tip of the camera and the camera itself used both freehand by the surgeon and in stabilised positions using a robotic laparoscopic controller (Freehand Laparoscopic Controller, Freehand, Surrey, UK) during ICG examination. Relative camera-to-target positions were calculated by moving the camera tip to recognisable locations in the rectum (e.g. distal and proximal healthy tissue regions as well as the tumour site as origin) after introduction through a standard transanal minimally invasive surgery (TAMIS) setup. Temporospatial data from the Aurora field generator, in combination with extracted time–fluorescence curves from three geographically distinct landmarks within the resulting NIR fluorescence video, were used to demonstrate distance–intensity relationships as well as reflect camera movement. Indiscriminate, rapid movement of the camera and the impact such use has on fluorescence intensity appearances was demonstrated by comparing fluorescence intensity tracking at the tumour and camera movements recorded by the EM tracking system. Normalisation as a technique to negate distance–intensity difficulties was demonstrated using this setup by comparing the time–intensity curve upslopes (i.e. ICG inflow) of two healthy regions of tissue with EM-calculated distance discrepancies. Potential mathematical solutions to movement issues were examined including smoothing of time–fluorescence curves using a Savitzky–Golay filter (to facilitate peak detection within fluorescence curves).

### Image presentation challenges

One video of a rectal cancer was chosen as a demonstrator of the potential for sample error in user-dependent ROI selection, akin to that seen in traditional biopsy, by comparing fluorescence curve outflow milestones of multiple tracked ROIs within a malignant lesion to each other, as well as to healthy control tissue within the same patient video.

### ML methods challenges

Factors pertinent to the data collection and analysis of ICG time–fluorescence curves utilising ML methods were assessed including optimal methods of automated data extraction from time–fluorescence curves as well as using methods to navigate missing data points whilst maintaining discriminatory classification performance (here, accurate healthy vs unhealthy tissue categorisation)*.* For this, at least one healthy control and one lesion tracing (either benign or malignant) were generated for each video to permit subsequent fluorescence curve milestone extraction [8, 11]. ROIs with missing downslope milestones (e.g. shorter duration videos) were calculated by imputing missing data points. K-nearest neighbour was used to impute within training sets with missing computed values compared to other known values within the set. Fitting to an exponential curve was used to impute missing values in testing curves to simulate real clinical practice where only a single video will be tested at any one time and therefore cannot be compared to ‘neighbours’. Following identification of a robust automated feature extraction methodology, AutoML (auto-sklearn) was used to identify an optimised classifier by searching over all possible hyper-parameters for a large range of known classifiers to choose the best candidate based on area under the receiver operating characteristic curve (AUC-ROC). Training–testing was performed using fivefold cross-validation with all available ROIs with an 80:20 training: testing split without holdout. ‘Time to first peak’, ‘Time ratio’ and ‘Downslope 10 s after peak’ were included in all classification combinations. Increasing durations of downslopes were incrementally added to ascertain the benefit of increased duration of tracking on performance. Classification was performed using two-way (cancer vs not cancer) as well as three-way (cancer vs benign tumour vs healthy control) splits. Finally, to investigate imputation as a method to replace missing values, all recorded results for downslope values beyond 10 s were removed (i.e. simulating a scenario where only data 10 s from the peak of inflow were available for all 37 patients) and replaced via imputation with the classification then re-run for comparison to the original results.

## Results

**Fig. 1:**
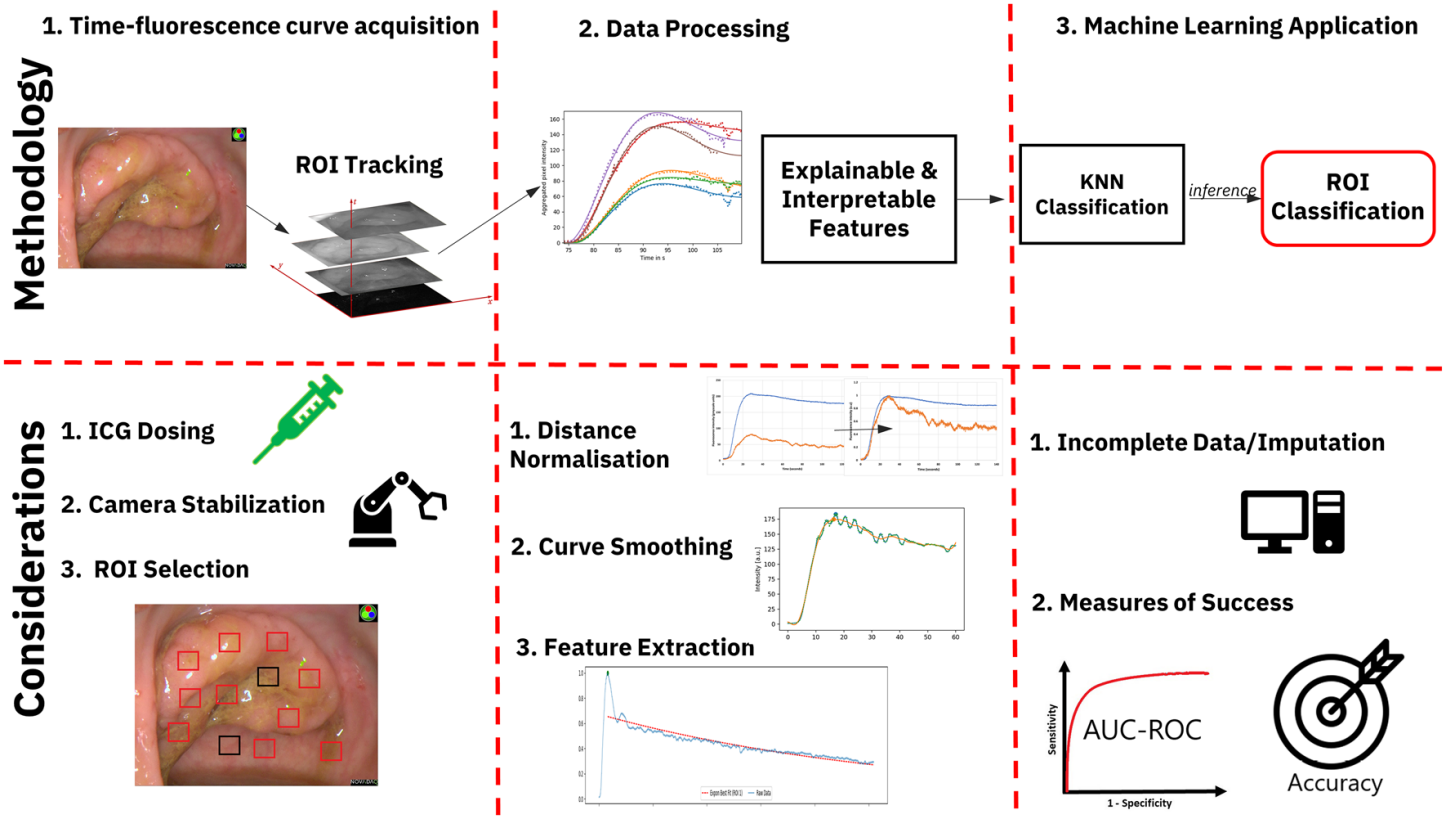
Diagrammatic representation of deterministic workflow process step components involved in ICG quantification, analysis and classification including important points defined for address at each step

Clinical, technical and technological challenges for appropriate application of ML (see Fig. 1) were commonly seen across the clinical series for both rectal and liver lesions and prompted evolutions in both acquisition protocols and post-acquisition data processing.

### Video acquisition

**Fig. 2:**
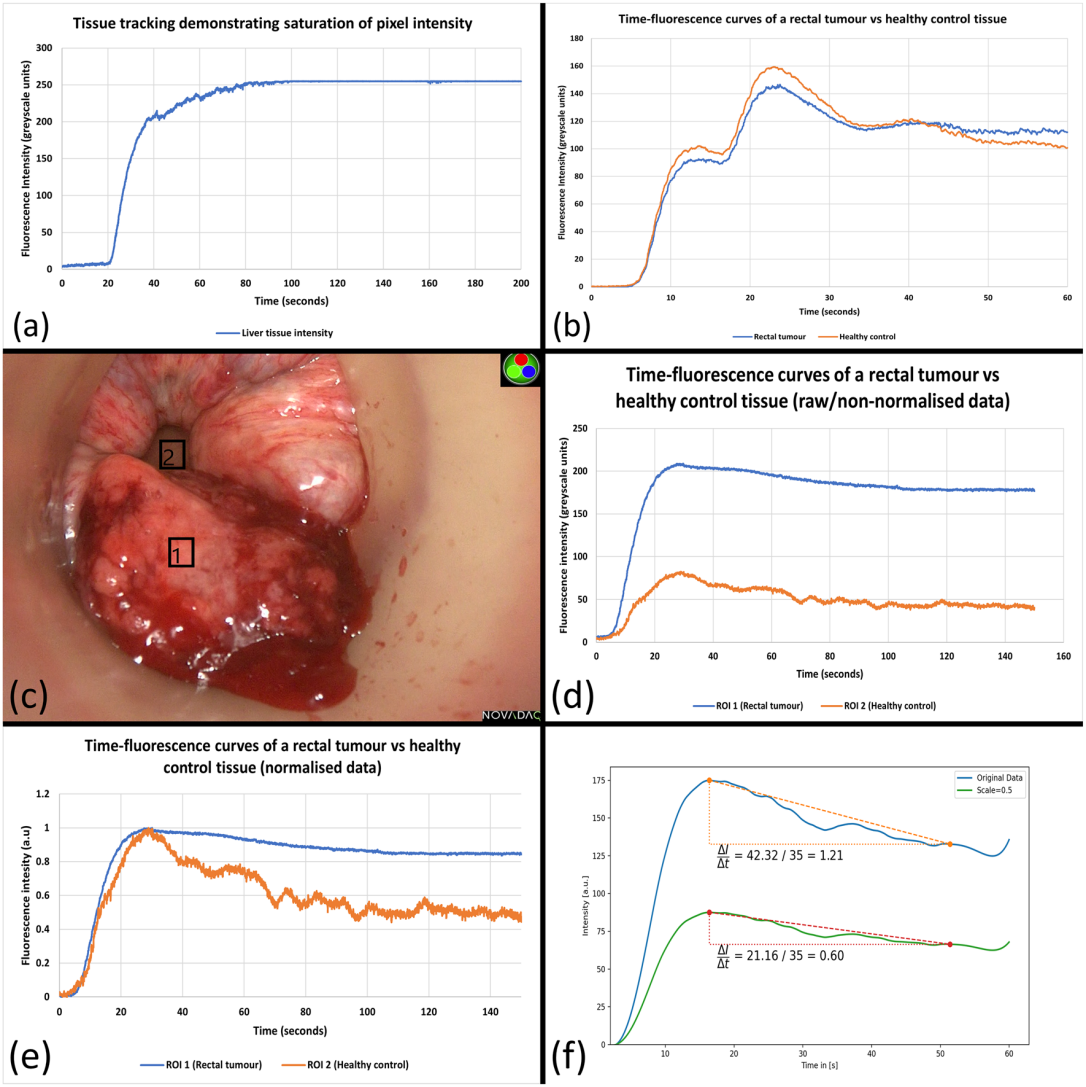
Compound image demonstrating important encountered challenges with methodological corrective counters for ICG quantification using extracted time–fluorescence curves from liver and rectal ICG fluorescence videos. (**a**) Time-fluorescence curve with signal saturation due to excessive dosing resulting in a persistent plateau in healthy liver tissue. (**b**) Time-fluorescence curve from a rectal lesion video with first peak at ≈ 15 s and second peak at ≈ 25 s due to ‘double bolus’ ICG push administration. (**c**) Image of a rectal cancer undergoing ICG assessment with transanal access platform in situ including supervised box annotations—ROI 1 represents rectal cancer tissue whilst ROI 2 represents distant control healthy tissue. (**d**) demonstrates large differences in tracked tissue fluorescence intensities (ROI1 vs RO2) as a result of distance discrepancy with fluorescence intensity is tracked dynamically from c. (**e**) Normalised time–fluorescence curves from the raw signal shown in image d. (**f**) Artificially generated graphs of two time–fluorescence curves identical re curve shapes but differing re absolute intensity values. Outflow slopes are also shown

Fixed dose ICG signal acquisition of rectal cancer utilised a wide range of the available greyscale spectrum on post-acquisition fluorescence curve analysis (pixel intensity range: 0–254.38 greyscale units across all 37 videos) without saturation. On the other hand, dosing issues related to signal saturation in healthy liver tissue, early in the series, were observed in two videos assessing CRLMs at 0.1 mg.kg ICG concentration. Subsequent reduced dose (0.05 mg/kg) imagery avoided such plateaus occurring (Fig. 2a). In the rectal tumour cohort, one “double peak” (Fig. 2b) occurred attributable to ICG dosing in two pulses.

### Distance–intensity relationships and movement

Time–intensity curves created from a rectal polyp video with a visible discrepancy in NIR camera-lesion distance (near) and healthy control (far away) are shown in Fig. 2. The raw (non-normalised) curves in this case show large differences in peak intensity readings (209.42 greyscale units vs 82.82 greyscale units) addressable by curve normalisation. The fallibility of comparing slope-based curve milestones between tissue fluorescence curves of raw/non-normalised data is shown in Fig. 2f using two artificially generated, identically shaped curves with different absolute intensity values (simulating the distance discrepancy seen in 2c and 2d).

**Fig. 3:**
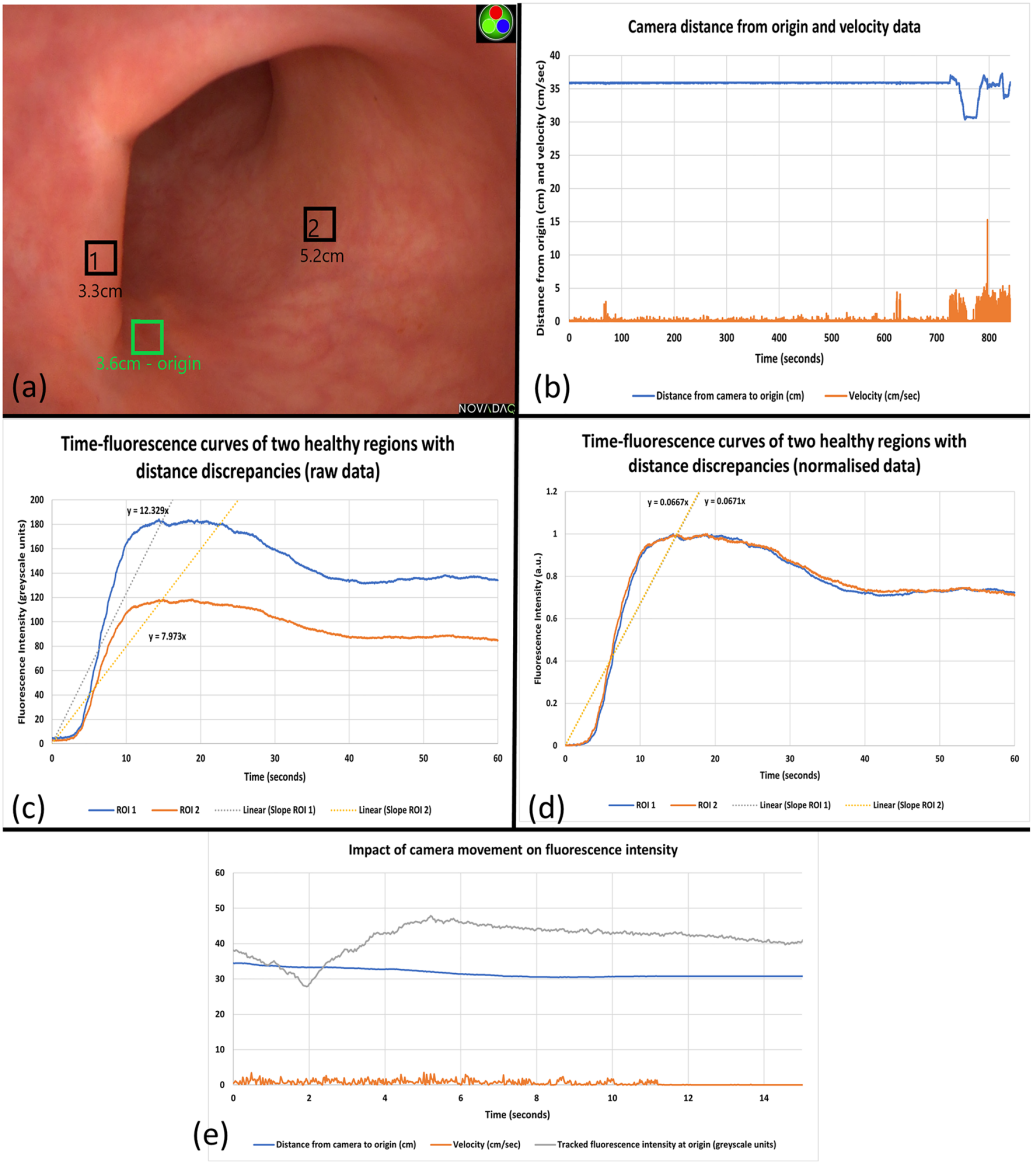
Distance, intensity and velocity data generated using an electromagnetic tracking system with its sensor probe attached to the tip of the camera and introduced into the patient with a rectal cancer following neoadjuvant therapy via a transanal access platform during examination under anaesthesia (EUA). (**a**) White light view of the rectum with regions of interest and their calculated distance to the camera tip displayed. (**b**) Distance, movement and velocity data of camera movement during the EUA and subsequent ICG assessment (note camera held with robotic camera holder until ≈720 s. (**c**) Raw time–fluorescence data achieved via tracking pixel intensity data of the regions indicated in the white light image with slope to peak intensities shown. (**d**) Appearance of these same regions after normalisation of intensity and upslope re-analysis. (**e**) Impact of camera movement on tracked fluorescence intensity showing rapid increase in detected fluorescence with inward movement of the camera toward the target tissue

**Fig. 4:**
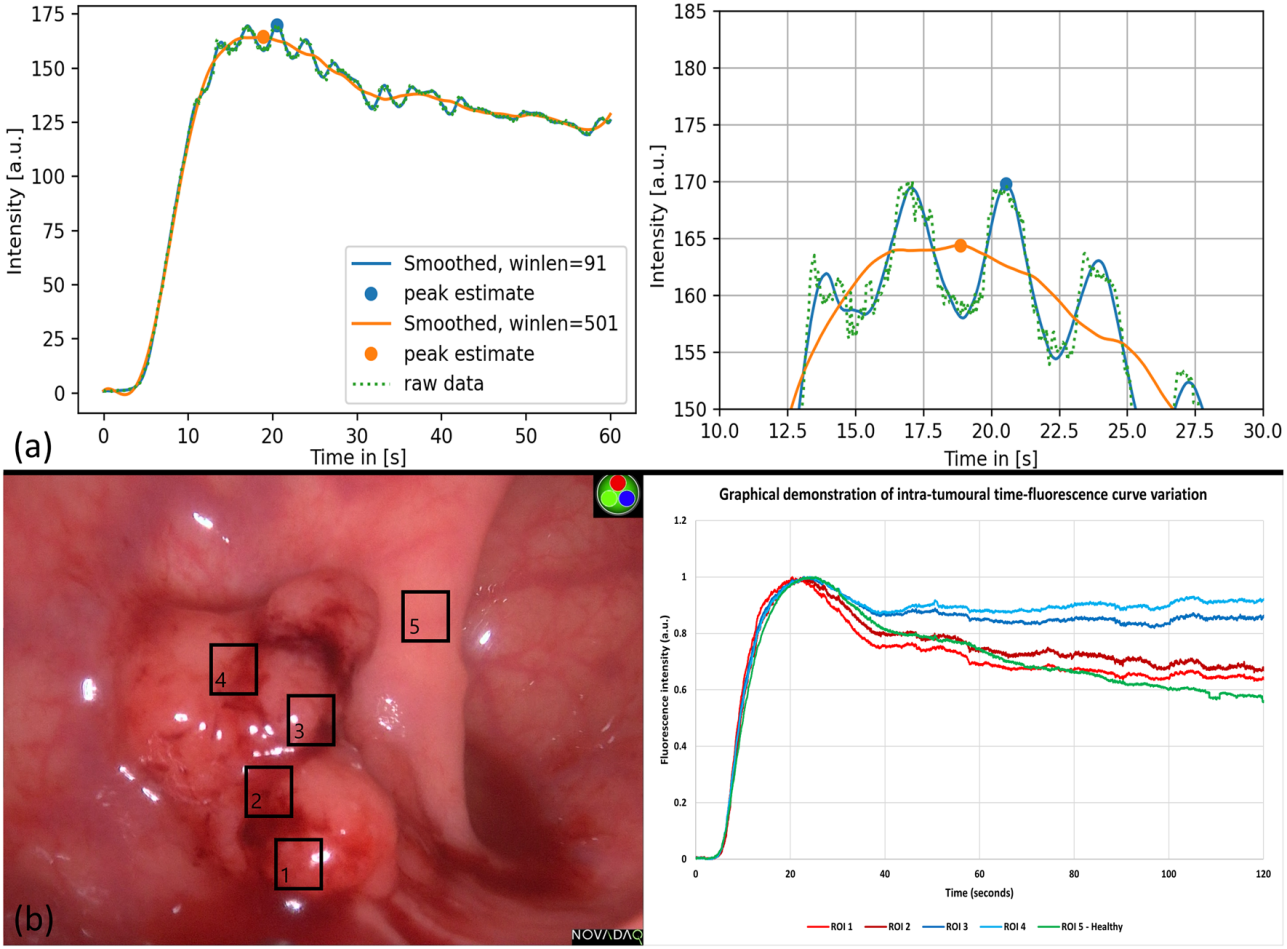
Compound image demonstrating smoothing (to mitigate signal oscillation) and intra-tumoural perfusion heterogeneity, (**a**) time–fluorescence curve from a rectal cancer with smoothing performed using a Savitzky–Golay filter, (**b**) white light view of a rectal cancer with time–fluorescence curves extracted from the ROIs marked

Camera stability over a prolonged period (11 min of ICG inflow/outflow) is demonstrated with a maximum camera movement of 2.15 mm over this time (Fig. 3b). Time-fluorescence curves of two highlighted healthy ROIs are shown in Fig. 3 with the impact of normalisation to allow comparison between the regions highlighted. Indiscriminate camera movement resulted in smooth tracing disruption previously seen with camera stabilisation (Fig. 3e) with a late tissue outflow increase in tracked fluorescence intensity being seen with camera movement toward the origin time–fluorescence curve oscillations (noise) successfully smoothed via a Savitzky–Golay filter (available from the open-source Scipy Python Package[21]) enabling peak detection (Fig. 4a).

### Digital biopsy sampling effects

Selecting only two intralesional ROIs yielded comparable outflow slopes to a healthy area (− 0.0065 and − 0.0053 vs − 0.0065). Once intralesional ROI sampling was increased to four regions, curves delineated significant fluorescence outflow variations vs benign tissue (ROI 3/4 outflow slopes = − 0.0013/− 0.0008, respectively).

### Machine learning data classification challenges

To develop and assess the tissue classifier and its features, curve milestones extraction from 251 ROIs across 37 rectal tumour videos was performed *(see supplementary table 1 for patient demographics and lesion data)*. Fluorescence time-series data were populated and output in comma separated values (CSV) files and curve milestones including upslope, time to peak and downslope were successfully extracted using open-source signal processing algorithms from the Scipy Python Package. All included ROIs had peak detection successfully performed with complete datasets then obtained up to 100 s of downslope. Missing downslope values were imputed for 11, 72 and 106 ROIs at downslopes of 200, 300 and 400 s, respectively. An ensemble composed of multiple *K-nearest neighbours* was identified as the best classifier based on AUC-ROC results (see Table 1). An excellent AUC-ROC score (0.92) was achieved on 2-way classification for all 251 ROIs when all downslope values to 400 s obtained during tracking were included (with imputed values where videos were of insufficient length). This result was improved further when three-way classification was performed (AUC–ROC = 0.93). Inclusion of downslope at just 200 s after peak resulted in an AUC-ROC > 0.9 for both two- and three-way classification. Analysis of the classification features showed ‘time to first peak’ as the most important feature followed by kurtosis and then downslope 200, 100, 50 and 10 s, respectively. Higher AUC-ROC scores were obtained when values were imputed out to 400 s.

**Table 1:** Results of two- and three-way classifications (including actual extracted features as well as imputed inference values) of all 37 included rectal tumour cases in this series. AUC-ROC values > 0.8 = good. > 0.9 = excellent

Two-way classification (cancer vs not cancer)		
Feature added (downslope after peak)	AUC-ROC (complete data)	AUC-ROC (imputed values)
50 s	0.805	0.799
100 s	0.881	0.856
200 s	0.888	0.852
300 s	0.912	0.856
400 s	0.920	0.872

Three-way classification (cancer vs benign tumour vs healthy control)		
Feature added (downslope after peak)	AUC-ROC (complete data)	AUC-ROC (Imputed values)
50 s	0.812	0.839
100 s	0.899	0.886
200 s	0.907	0.875
300 s	0.910	0.898
400 s	0.931	0.891

## Discussion

Given the fallibility in human interpretation of dynamic ICG perfusion angiograms [22], quantification and computer-assisted interpretations, including ML, have been explored for the purpose of providing more objective, and potentially more detailed, fluorescence signal interpretation with determination of confidence levels. Application of such methods to real-world data and ideally, of course, in real-time, however, will require a variety of considerations to be addressed as shown in this work. Here, we have shown how such concerns can be factored into a capable CV-ML method with consistent precision.

ICG dosing remains a topic of considerable discussion with wide variations in both weight-based and fixed dosages being reported for colorectal and hepatobiliary assessment. The 0.25 mg/kg rectal lesion dosing described in this paper utilised the entire available intensity scale without saturation (avoiding a false-intensity plateau and compromised data analysis). Conversely, a smaller dose (0.1 mg/kg) in the liver lesion cohort resulted in early saturation of pixel intensity due to liver hepatocyte concentration and excretion of ICG and highlights the importance of attention to dose and tissue type when quantifying ICG administered intraoperatively. Antecedent dosing hours-days preoperatively as described by others for CRLM localisation depends on complete washout from healthy tissue and retention in/near malignant tissue over the prolonged preoperative period of time providing high tumour-to-background ratio at the time of interrogation, meaning high ICG doses can be used successfully. Whilst intravenous ICG administration method (especially central vs peripheral line) is discussed in the literature, the need for rapid, single bolus administration when using quantitative ICG fluorescence angiography is demonstrated here and needs to be clearly understood by the administrator. Deviations may result in false flow peaks that possibly derail automated peak detection software and ML algorithms.

The incorporation of absolute intensity as a feature for analysis also needs address. Whilst normalisation of data to a maximum peak brightness across all ROIs results in the loss of absolute intensity values as a potential feature in any subsequent computation, this study demonstrates the impact of using raw data to compute slope-based parameters. Furthermore, this permits the comparison of tissue fluorescence curves without requiring both tissues to be in the centre of the screen and at similar distances which is not always clinically possible. Although it may be possible to adjust for such distance–intensity relationships automatically within future NIR systems that incorporate distance data (like that we describe using EM tracking fields system for instance), normalisation of raw data currently provides a useful alternative yielding excellent and reproducible results.

Movement either at tissue (e.g. peristalsis, respiration, patient heaving, etc.) and/or at operator-held camera level can in theory be problematic. Use of a robotic camera holder as described here results in minimal camera movement although similar results can be achieved by a human operator steadying the view by careful attention to the video display especially for focussed time periods. Large amounts of movement (> 5 cm of movement over 5 s) can result in false signal detection (e.g. an apparent increase in intensity during outflow despite no further ICG administration); however; all videos utilised in the classifier described were obtained with a handheld camera approximately 3–8cms from the target (i.e. standard TAMIS working distance) with any resulting small deviations being adequately managed through curve smoothing.

Tissue biopsy sampling errors, due to tumour heterogeneity, is a significant clinical problem with high false-negative rates occurring in larger polyps [23, 24]. Digital biopsy in the fashion described may also suffer from the same phenomenon; however, it more easily affords the opportunity to sample large areas of lesions as demonstrated. Care must be taken to analyse as large a tissue area as possible; however; limited user-selected ROI tracking remains prone to under-representation. Next step evolution of this work includes real-time image stabilisation with synchronous white light surface feature tracking and whole screen equivalent pixel intensity extraction providing full screen characterisation with heat map display. Such an approach would also permit tumour margination via such automated classification.

Time-series feature extraction and analysis, including imputation to deal with missing data as may arise due interruption of the continuous field of view observation by movement or indeed surgical instrumentation intrusion, is demonstrated to yield consistent, positive results across a relatively large cohort of patients. The methods of data management (e.g. curve milestone extraction, curve smoothing, etc.) detailed within this manuscript, whilst applied to ROI data extracted from the described open-source fluorescence intensity tracker, can be applied similarly to other software programmes (such as the Fluorescence Tracker App by MATLAB available at www.mathworks.com) as well as to other curve milestones which may be more relevant in other applications (such as colonic intestinal perfusion assessment) [20]. We chose AUC-ROC (rather than ‘straight’ accuracy) to report the results of the classifier as AUC-ROC is the probability that the model ranks a random positive example more highly than a random negative example [25]. The resulting performance figure is, therefore, an indication of how much better a classifier is at correctly classifying than misclassifying which is not so clear from just accuracy rate alone. High AUC-ROC scores are obtained at 200 s with only small improvements achieved with tracking beyond this timeframe. In the case of missing values, however, imputation of downslope values up to 400 s improved the AUC-ROC result and suggests that should imputation be required for missing data, it should be done for longer periods of time if possible. It should be noted that although the results of imputation, when performed on all ROIs, dropped below 0.9, these results were obtained by simulating a scenario where tissues were tracked for only 10 s beyond their peak intensity. This was to simulate an extreme scenario (less than a minute of actual tracking per video) and was done to demonstrate feasibility of this approach in such circumstances. It is likely, however, that some data will be lost intermittently during surgery (camera movement, patient movement, etc.) and this method of imputation demonstrates a robust method for dealing with such occurrences. Whilst this technique is demonstrated here for cancer characterisation, the methodology can be applied too to other time-curve applications. Finally, the classifier’s ability to maintain “excellent” results during three-way classification “benign dysplasia vs healthy vs cancer”, which is inherently more difficult than two-way “cancer vs benign” discrimination, is encouraging for future further sub-classification work and suggests that with increasing datasets, discrimination by dysplasia type (low vs high grade) and T stage will likely be possible as well as in other important clinical scenarios such as rectal lesion interrogation post-neoadjuvant therapy.

Limitations in this study relate to the specific nature of the dataset although similar considerations likely also apply to tissue perfusion signal capture for other dynamic-based indications (e.g. intestinal or flap assessment). However, open assessments (whether extracorporealised viscera or plastic surgery operations) are most often done by stabilised cameras and/or systems with bigger camera heads enabling more intense illumination and signal sensing. Also, the study only involved one type of NIR system and other systems are known to differ in their signal capture and display. However, the work identifies the important considerations to be considered and all systems will need specific user guidance in similar respect. Such study of alternative commercial systems especially with regard to distance–intensity is already ongoing. The retrospective nature of the video analysis is also a potential limitation although consistently high results have been demonstrated with several diverse methods of time–fluorescence curve analysis both based on feature extraction and classification as well as profile fitting based on biophysical modelling and subsequent classification [10, 11].

In conclusion, whilst ICG quantification is not readily available in most clinical NIR systems currently, time-series quantification, and subsequent analysis, can be performed with excellent results with adherence to important intraoperative physical and technical recommendations and purposeful post-processing of data. Best presentation of the dynamic imagery for interpretation by machines is as important from this perspective as image display is for human observer interpretation. Whilst some technological advancement is needed to enable this seamlessly in real time, the computational trajectory seen over the last few decades should give confidence that evolved processing capability can encompass all such aspects.

## Supplementary Information

Below is the link to the electronic supplementary material.

Supplementary file1 (DOCX 14 KB)
