# Supplementary material for: Clinical application of machine learning and computer vision to indocyanine green quantification for dynamic intraoperative tissue characterisation: how to do it
Source: Surg Endosc. 2023 Mar 9;37(8):6361–70. doi: 10.1007/s00464-023-09963-2 (PMC10338552; doi:10.1007/s00464-023-09963-2)
Supplement: Supplementary file 1 — Supplementary file1 (DOCX 14 KB) [file 464_2023_9963_MOESM1_ESM.docx]

*Supplementary Table 1: Patient demographics and lesion data for all 37 included rectal cases.*

| **Rectal Lesion Patient Characteristics** | **N=37 cases** |
| --- | --- |
| Male: Female | 24 (65%):13 (35%) |
| Age (Mean ± Std dev) | 70±10.18 |
| Benign: Malignant | 13 (35%):24 (65%) |
| Neoadjuvant Tx at time of ICG assessment | 5 (14%) |
| **Final Pathology** | |
| Low Grade Dysplasia | 10 (27%) |
| High Grade Dysplasia | 3 (8%) |
| T1 | 4 (11%) |
| T2 | 11 (30%) |
| T3 | 6 (16%) |
| T4 | 3 (8%) |
